# Is previous radical prostatectomy an adversity for laparoscopic total extraperitoneal approach for inguinal hernia repair? A propensity score case matched study

**DOI:** 10.1007/s10029-025-03363-2

**Published:** 2025-05-23

**Authors:** İsmail Ahmet Bilgin, Nur Ramoglu, Volkan Ozben, Orkun Harun Çebi, Omer Burak Argun, Tunkut Salim Doganca, Ali Rıza Kural, Bilgi Baca, İsmail Hamzaoglu, Tayfun Karahasanoglu

**Affiliations:** 1 Department of General Surgery, School of Medicine, Acibadem Maslak Hospital, Acibadem Mehmet Ali Aydinlar University, Istanbul, Türkiye; 2 Department of General Surgery, School of Medicine, Acibadem Mehmet Ali Aydinlar University, Istanbul, Türkiye; 3 Faculty of Health Science, İstanbul Kent University, İstanbul, Türkiye; 4 Department of Urology, Acibadem Taksim Hospital, İstanbul, Türkiye; 5 Department of Urology, School of Medicine, Acibadem Mehmet Ali Aydinlar University, Istanbul, Türkiye; 6 Department of General Surgery, School of Medicine, Acibadem Altunizade Hospital, Acibadem Mehmet Ali Aydinlar University, Istanbul, Türkiye

**Keywords:** Inguinal hernia, Laparoscopic TEP repair, Previous radical prostatectomy, Outcomes

## Abstract

**Introduction:**

Although laparoscopic total extraperitoneal (TEP) procedure has gained wide acceptance for inguinal hernia repair, there is still debate on the optimal technique in patients with a history of previous radical prostatectomy (RP). We aimed to evaluate the feasibility and safety of laparoscopic TEP in patients with a previous history of RP using a propensity score case-match analysis.

**Methods:**

This study included male patients undergoing laparoscopic TEP repair between 2013 and 2024. According to the RP status, patients were case-matched based on age, BMI, ASA score, site of hernia and the year of surgery. A total of 162 patients were matched in a 1:5 ratio. The RP and non-RP groups were compared with respect to perioperative outcomes.

**Results:**

The RP and non-RP group included 27 and 135 patients, respectively. The rate ofconversion to transabdominal preperitoneal repair (11.1%) or open surgery (14.8%) was significantly higher in the RP group (p<0.001). The RP group had longer operative times (160±57 vs. 94±38, p<0.001). The postoperative complication rates (7.4% vs. 6%), postoperative pain scores, length of stay (1.6±0.9 vs 1.2±0.9 days), time to return to daily life (2.9±1.8 vs 2.6±3.0 days), readmission (3.7% vs 0.7%), long-lasting pain (14.8% vs. 11.8%) and recurrence (0% vs 3.2%) were similar in both groups (p>0.05).

**Conclusion:**

Laparoscopic TEP inguinal hernia repair in patients with a history of RP is feasible and safe with a similar morbidity profile but an increased conversion rate and operative time compared to those with no history of RP.

## Introduction

Currently, laparoscopic total extraperitoneal (TEP) and transabdominal preperitoneal (TAPP) approaches are favored over conventional open inguinal hernia repair in general surgery practice. This preference arises from the recognized advantages of minimally invasive surgery, including reduced pain and faster recovery [1]. TAPP is a commonly performed technique due to its shorter learning curve and greater experience among surgeons [2]. On the other hand, the TEP technique has the advantages of less risk of visceral injuries, scrotal edema, port site hernias and reduced postoperative pain, all of which have been reported be associated with better patient satisfaction when compared to the TAPP technique [3, 4].

Nevertheless, minimally invasive inguinal hernia repair presents some challenges in patients with a history of previous lower abdominal surgery. Previous surgical procedures can result in adhesions and scar tissue that can disrupt the dissection planes in the operative field complicating laparoscopic inguinal hernia repair, especially making the TEP approach difficult [5]. Radical prostatectomy (RP) is one of the commonly performed lower abdominal surgeries in males. It is also known to increase the likelihood of developing inguinal hernias [6]. RP creates its own set of unique challenges, particularly concerning dissection in the preperitoneal area during TEP repair [7]. Consequently, open surgery is often the preferred option for this subset of patients in most clinics [5, 8]. On the other hand, some studies indicate that the TEP approach is both feasible and safe even in patients with a history of RP [7, 9] whereas others recommend the use of TAPP technique in such patients [10].

Therefore, we aimed to evaluate the feasibility and safety of laparoscopic TEP inguinal hernia repair in patients with a history of previous RP in this study.

## Methods

### Study design

A total number of 797 patients with the diagnosis of inguinal hernia who underwent hernia repair in a single center, Acibadem Maslak Hospital, between January 2013 and December 2024 were evaluated. The study was designed as a retrospective propensity score case-match study. Informed consent was taken from each patient. The study protocol was approved by the Ethics Committee of Acibadem Mehmet Ali Aydinlar University (ID number: 2023-07/257).

The inclusion criteria consisted of male patients over 18 years of age with unilateral or bilateral inguinal hernia who underwent laparoscopic TEP repair. Emergency operations, recurrent cases, TEP procedure with any additional surgical interventions, and patients with any previous lower abdominal surgeries other than RP were excluded.

**Fig. 1:**
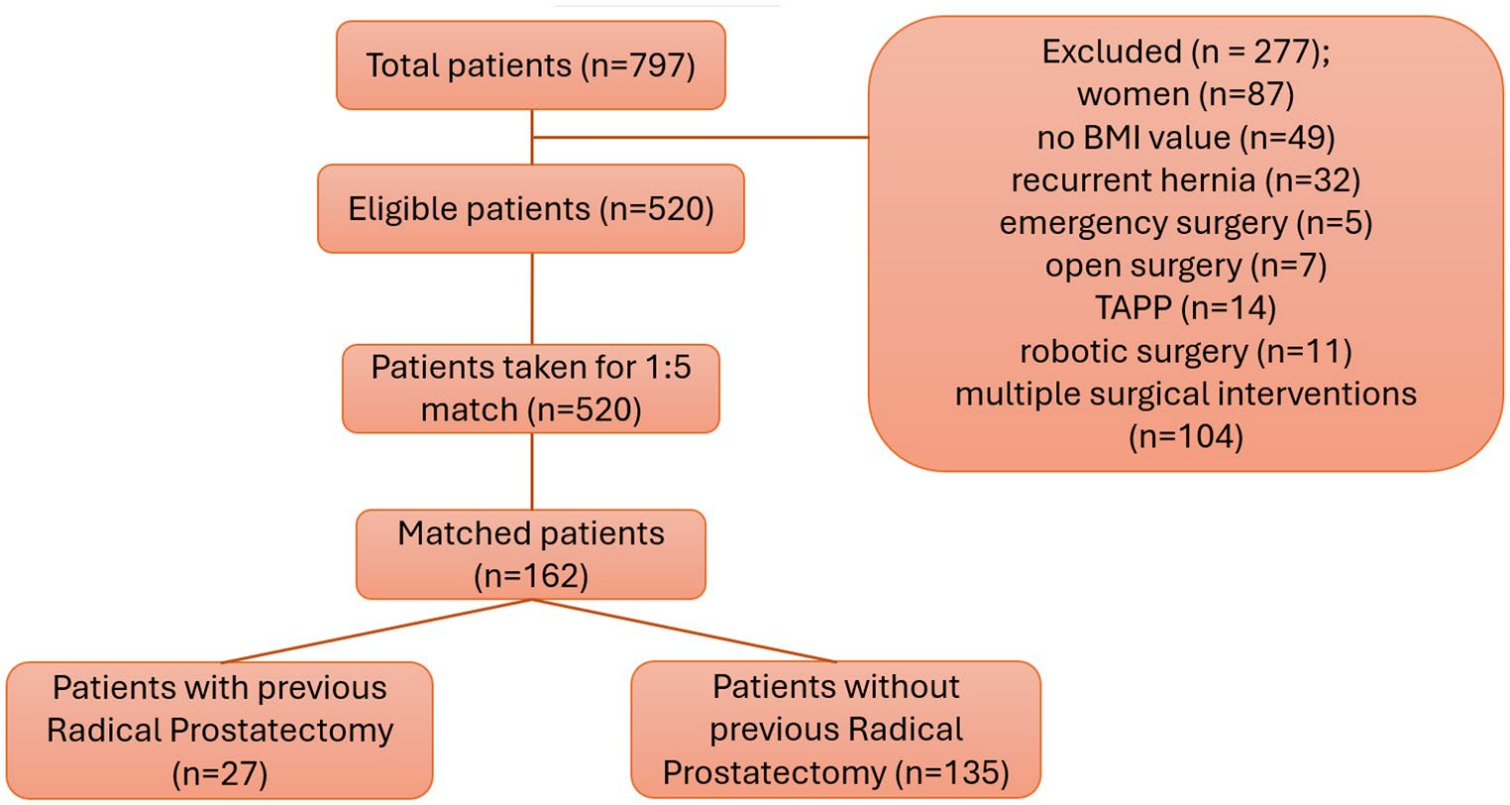
Consort diagram of patients undergoing laparoscopic TEP inguinal hernia repair

After exclusion, 520 eligible patients were selected for case matching. The matching criteria were age, body mass index (BMI), American Society of Anesthesiologists (ASA) classification, site of herniation (unilateral or bilateral), and the year of surgery. Utilizing a matching ratio of 1:5, 162 patients were included for the final analysis. A flowchart detailing the patient selection process is presented in Fig. 1.

Data collected included patient demographics, ASA score, BMI, operative approach for previous RP (open, laparoscopic or robotic), prior radiotherapy, time elapsed since RP, hernia size, hernia type, operative time, intra- and postoperative complications, conversions, drainage use, postoperative numeric rating scale (NRS) score for pain within the postoperative first 24 h, duration of hospital stay, readmissions, time to return to daily life, postoperative long-lasting pain, chronic pain, restriction in movement, and hernia recurrence. Hernia size was classified according to the European Hernia Society (EHS) groin hernia classification [11]. Operative time was defined as the time from the first skin incision to the final closure of the skin incisions. Conversion was defined as conversion of the TEP procedure to either TAPP or open surgery. Pain severity within the postoperative 24 h was evaluated with a NRS score (0, no pain; 10, very serious pain). Pain severity of 1–4 was considered as mild, 5–7 as moderate, and 8–10 as severe pain [12]. Pain which continued for the first month, disabling the patient from doing daily activities was accepted as long-lasting pain. Chronic pain was defined as new-onset pain for more than 3 months after surgery. All the recurrences were identified through clinical visits and were confirmed with ultrasonography.

The clinical characteristics and outcome parameters, including operative time, conversions, complications, pain scores, and recurrence rates were compared between the propensity score case-matched RP and non-RP groups. The main outcome parameters were conversions, complications and recurrences. A subgroup analysis was also conducted to evaluate perioperative outcomes according to the previous operative approach (robotic versus open) in the RP group.

### Surgical technique

A standardized TEP inguinal hernia repair was performed in all the patients. All the patients were treated by a single operative team with the same level of experience of more than 10 years in laparoscopic TEP procedure. The decision to perform TEP repair was based on the high experience of our surgical team with this technique and the accumulating literature data which shows the feasibility of TEP procedure for the treatment of unilateral or bilateral inguinal hernias after RP.

Under general anesthesia, a 1-cm transverse skin incision was created just inferior to the umbilicus off the midline and carried down sharply to the level of the anterior rectus sheath. The anterior rectus sheath was incised transversely off the midline exposing the rectus abdominis muscle. The rectus abdominis muscle was retracted laterally to reveal the posterior rectus sheath. For the initial access, a balloon spacemaker was inserted into the preperitoneal space towards the symphysis and inflated 8 times. In patients with previous RP, we especially placed a 10-mm 0-degree laparoscope through the spacemaker to stay at low inflation, reducing inadvertent bleeding due to scar formation. Then, a Hasson cannula was placed into the space which was insufflated with CO_2_ to 10–12 mmHg. A 10-mm 30-degree laparoscope was inserted through the cannula and two additional 5-mm trocars were placed in the lower midline abdomen under direct vision: one trocar above the symphysis pubis, and the other in the middle between that one and the 10-mm trocar. First, we performed inspection of the inguinal space, identifying the inferior epigastric artery which is the most important landmark. Dissection started laterally close to the anterior abdominal wall and continued towards the lateral abdominal wall, anterior superior iliac spine and finally the space of Bogros and Retzius. Structures of the spermatic cord were prepared, and the herniated sac was separated from these structures and reduced in both incomplete and complete (inguinoscrotal) hernias. After dissection was completed, a 15 × 10-cm 3D mesh was introduced through the umbilical trocar and placed covering three anatomical sites: the Hasselbach’s triangle, deep inguinal ring, and femoral area. The mesh was routinely fixed to the superior pubic ramus periosteum, linea alba and anterior abdominal wall using 5–6 titanium tacks. Suction drains were placed in extraperitoneal area if there was a large defect, increased risk of bleeding or seroma formation in the case of direct hernias. Urethral catheter was not used in any of the cases.

### Postoperative care

The operation was usually planned with a one-day postoperative hospitalization period. Postoperative pain was controlled with standard analgesics, including non-steroidal anti-inflammatory drugs and paracetamol of the same dosage for each case. Discharge planning was standard with directions for full daily activities and a regular diet as tolerated. Postoperatively, the suction drain, if present, was generally removed on the first postoperative day and the patients were seen after 7 days in the outpatient clinic. The surgeon’s contact number was shared with each patient and the patients were advised to contact the surgeon about any postoperative issues regarding any complications, chronic pain and recurrences.

### Statistical analysis

Statistical analyses were performed using R Studio and R statistical software, with significance set at a 95% confidence level. A propensity score-matched analysis was conducted using MatchIt package [13]. Control patients were matched 5:1 to prostatectomy patients, considering age, BMI, ASA score, year of surgery, and herniation site (unilateral/bilateral) to minimize baseline differences between groups. Shapiro-Wilk test was used to evaluate normality. Because none of the continuous variables followed a normal distribution, all continuous variables were reported as medians with interquartile ranges, while categorical and ordinal variables were expressed as absolute and relative frequencies. Group differences in age, BMI, operation time, postoperative hospital stay, prior hernia surgery, conversion to open surgery, conversion to TAPP, complication rates, readmission rates, and return to daily activities were analyzed using the Mann-Whitney U test. Differences in ASA scores, unilateral/bilateral status, pain (NRS) within the first 24 h and the first month, and 30-day movement limitations were assessed using the χ2-square test.

## Results

The RP and non-RP groups included 27 and 135 patients, respectively. Demographics and clinical data are provided in Table 1. Overall, 36 patients had unilateral and 126 had bilateral inguinal hernias, corresponding to a total number of 288 hernia repairs in both groups. There were no significant differences between the two groups regarding age, ASA scores, BMI and the year of surgery due to the case-match design of the study. In the RP group, 7 (25.9%) patients had unilateral and 20 (74.1%) had bilateral hernia repairs (47 in total) whereas in the non-RP group, 29 patients (21.5%) had unilateral and 106 (78.5%) had bilateral hernia repairs (241 in total) (*p* = 0.8). There were no significant differences regarding the EHS classification of both right-sided and left-sided hernias between the groups.

**Table 1:** Comparison of clinical characteristics, intra- and postoperative outcomes between the groups

		RP group (*n* = 27)	Non-RP group (*n* = 135)	*P* value	
Age, years, mean ± SD		67.7 ± 10.7	66.9 ± 9.1	0.711	
ASA score, n (%)				0.327	
1		10 (37.0)	68 (50.4)		
2		17 (63.0)	66 (48.9)		
3		0 (0)	1 (0.7)		
BMI, kg/m^2^, mean ± SD		25.7 ± 2.7	26.1 ± 3.3	0.39	
Site of hernia, n (%)				0.8	
unilateral		7 (25.9)	29 (21.5)		
bilateral		20 (74.1)	106 (78.5)		
EHS classification of right-sided hernia, n (%) *				0.11	
class 1		13 (54.2)	43 (34.4)		
class 2		7 (29.2)	65 (52.0)		
class 3		4 (16.6)	17 (13.6)		
EHS classification of left-sided hernia, n (%) ^†^				0.52	
class 1		9 (42.9)	46 (41.8)		
class 2		12 (57.1)	55 (50.0)		
class 3		0 (0)	9 (8.2)		
Operative time, min, mean ± SD		160 ± 57.0	93.7 ± 37.7	< 0.001	
unilateral		137.1 ± 62.1	85.9 ± 43.3	0.075	
bilateral		168 ± 54.5	95.8 ± 35.9	< 0.001	
Conversions, n (%)				< 0.001	
TAPP		3 (11.1)	0 (0)		
open		4 (14.8)	0 (0)		
Intraoperative complications, n (%)		1 (3.7)	0 (0)	0.17	
Drain use, n (%)		12(44.4)	59 (43.7)	> 0.99	
Amount of drainage, ml, mean ± SD	100.4 ± 59.3		86.5 ± 52.2	0.44	
Postoperative pain score in 24 h, mean ± SD	2 ± 1.7		2.1 ± 2.0	0.76	
Postoperative complications, n (%)	2 (7.4)		6 (4.4)	0.62	
Hospital stay, days, mean ± SD	1.6 ± 0.9		1.2 ± 0.9	0.07	
Return to daily life, days, mean ± SD	2.9 ± 1.8		3.6 ± 3.0	0.11	
Long-lasting pain, n (%)	4 (14.8)		15 (11.8)	0.75	
Chronic pain, n (%)	0 (0)		0 (0)		
Movement restriction within the first month, n (%)	2 (7.4)		13 (10.2)	> 0.99	
Readmission, n (%)		1 (3.7)	1 (0.7)	0.31	
Recurrence, n (%)		0 (0)	4 (3.2)	> 0.99	
Follow-up time, months, mean ± SD		48.7 ± 39.8	50.8 ± 37.2	0.8	
Lost to follow-up, n (%)		0 (0)	8 (5.9)	0.19	

*ASA* American Society of Anesthesiologists, *BMI* body mass index, *EHS* European Hernia Society, *TAPP* transabdominal preperitoneal

* Data were unavailable for 3 right-sided hernias

^†^ Data were unavailable for 5 left-sided hernias

**Fig. 2:**
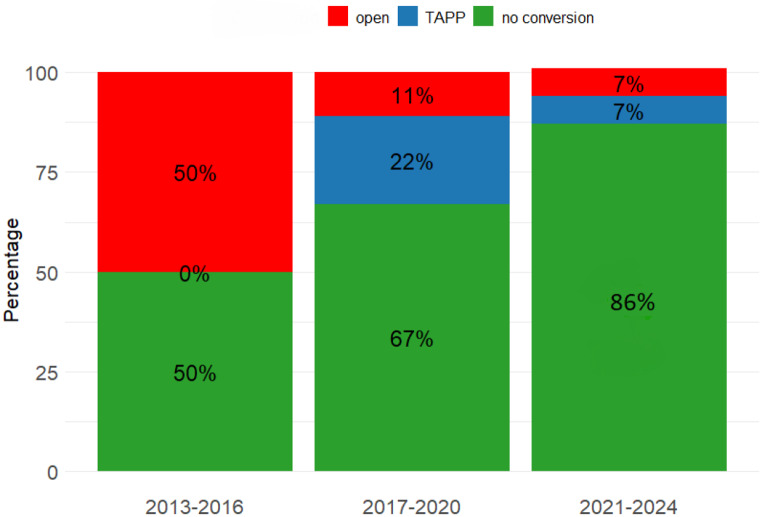
Conversion rates over the years in the RP group

The mean operative time was significantly longer in the RP group (160 ± 57.0 vs. 93.7 ± 37.7 min) (*p* < 0.001). However, when the mean operative time across the unilateral and bilateral cases was compared separately, it was not significantly different with respect to the unilateral cases (*p* = 0.075). The rate of conversion to TAPP or open surgery was significantly higher in the RP group (25.9% vs. 0%, *p* < 0.001). There was no conversion in the non-RP group while there were 7 converted cases in RP group. In the RP group, 3 (11.1%) cases were converted to TAPP, and 4 (14.8%) cases were converted open surgery. The reasons of conversion to open surgery were extensive adhesions due to preperitoneal scarring and a creation of large peritoneal defect which made further dissection impossible in the preperitoneal area. In other circumstances, conversion to TAPP was performed. Among these 7 converted cases, 6 cases (5 bilateral and 1 unilateral) required conversion for only one side and 1 case (bilateral) required conversion for both sides. As a result, 8 (17.0%) conversions occurred in a total number of 47 hernia repairs. In the RP group, further analysis revealed a remarkable decrease in the conversion rate from 50% in the years from 2013 to 2016, to 33% from 2017 to 2020, and finally to 14% from 2020 to 2024. Additionally, there was no conversion in any of the last 12 RP cases since 2022 (Fig. 2).

Intra- and postoperative complication rates were similar between the groups (intraoperative, 3.7% vs. 0%; postoperative, 7.4% vs. 4.4%, *p* > 0.05). Intraoperatively, bladder injury occurred due to severe adhesions in a patient in the RP group; laparoscopic primary repair was performed, and the patient was discharged without any further complications. Suction drainage tube was placed in 12 (44.4%) patients in the RP group and in 59 (43.7%) patients in the non-RP group (*p* > 0.999). Postoperatively, the RP group had hematoma in 1 patient and seroma in 1 patient; the cases were managed conservatively. The non-RP group had seroma in 3 patients, hematoma in 2 patients and severe inguinal pain in 1 patient; seroma was managed with needle aspiration in all the cases, cases with hematoma were followed up and severe inguinal pain responded well to nonsteroidal anti-inflammatory medication. No bowel, vascular, spermatic cord injuries or infections occurred in any group. The mean length of hospital stay was similar between the groups (1.6 ± 0.9 vs. 1.2 ± 0.9 days, *p* = 0.07).

**Fig. 3:**
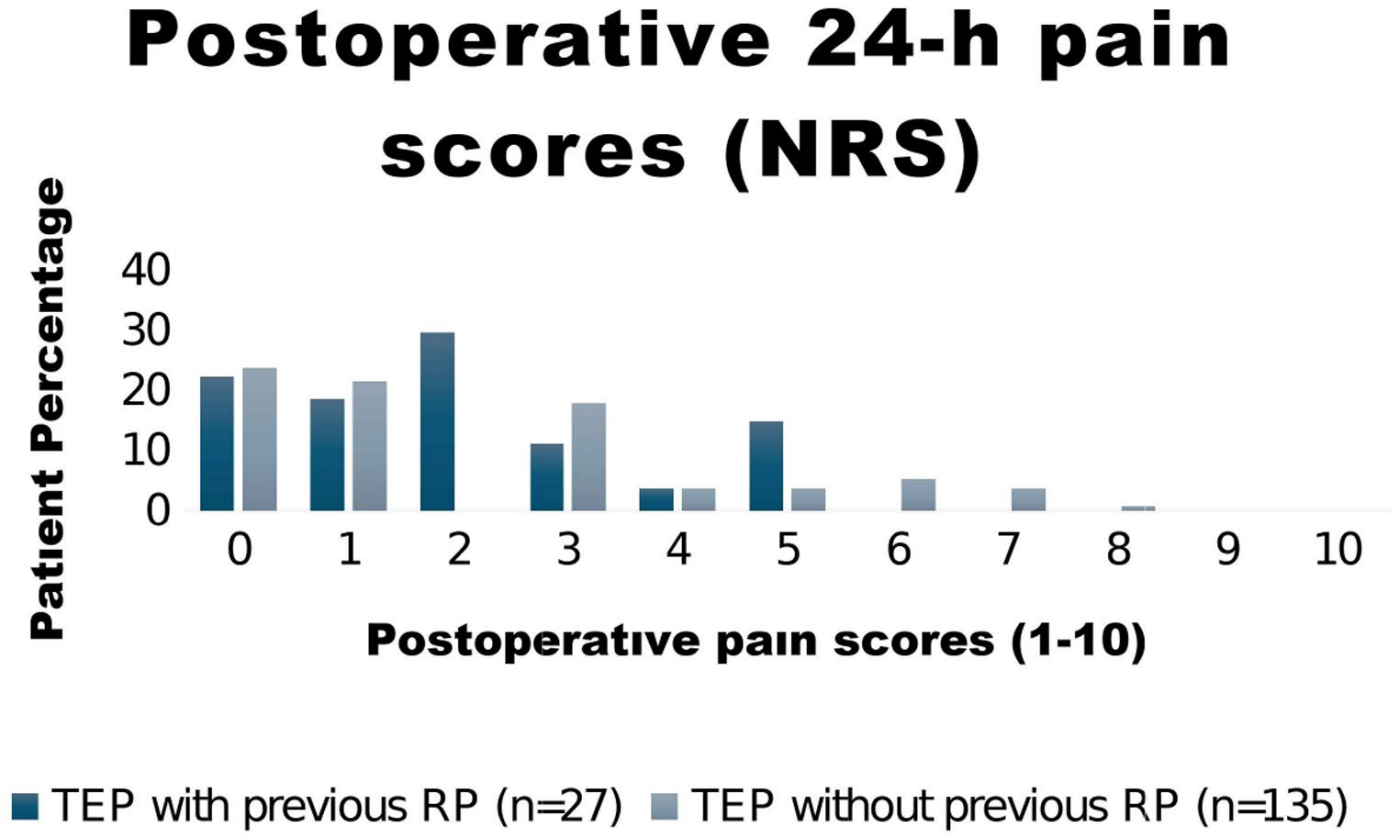
Postoperative 24-hr pain scores (*NRS* numerical rating scale)

Overall, 1 (0.01%) patient had severe pain, 21 (13.0%) patients expressed moderate pain, 102 (63.4%) mild pain and 38 (23.6%) patients had no pain in the postoperative first 24 h. With respect to the postoperative NRS pain scores, there was no significant difference between the groups (2 ± 1.7 vs. 2.1 ± 2, *p* = 0.76). Postoperative 24 h pain score comparison according to the scale is shown in Fig. 3. Regarding the mean time to return to daily activities, there was also no significant difference (2.9 ± 1.8 vs. 3.6 ± 3.0, *p* = 0.11). Only 2 patients, 1 in the RP group and 1 in non-RP group were readmitted to hospital (*p* = 0.31); the reasons for readmission were seroma formation in one patient and hematoma in the other. Within the first postoperative month, 2 (7.4%) patients in the RP group and 13 (10.2%) patients in the non-RP group expressed movement restriction (*p* > 0.99) and the reason for movement restriction was groin pain in all these cases. Within the first month, long-lasting pain occurred in 4 and 15 patients in the RP and non-RP groups (14.8 vs. 11.8%), respectively with no statistical difference between the groups (*p* = 0.75). None of the patients had chronic pain.

The mean follow-up time for RP and non-RP groups were 48.7 ± 39.8 and 50.8 ± 37.2 months, respectively. Eight patients were lost to follow-up in the non-RP group while none of the patients in RP group were lost to follow up. No recurrence occurred in RP group while 4 (3.2%) patients had recurrence in the non-RP group with no statistical significance (*p* > 0.99).

In the RP group, the median time interval between the prior radical prostatectomy and hernia repair was 40.5 (range, 3.3–230) months. Five patients received radiotherapy; 3 (11.1%) patients prior to hernia repair (the mean time elapsed since radiotherapy was 96.7 ± 42.2 months) and the other 2 (7.4%) patients after hernia repair due to prostatic cancer recurrence. In subgroup analyses, there were no correlations between time elapsed since prior radical prostatectomy or radiotherapy on intra- and postoperative complications, conversion rates, drain use, readmissions, pain and movement restriction in first month and recurrence rates (*p* > 0.05).

Regarding the type of surgical approach for RP, it was robotic in 21 (77.8%), open in 5 (18.5%), and laparoscopic in 1 (3.7%) patient. Excluding the laparoscopic case, comparison of the clinical characteristics and perioperative outcomes according to the previous robotic versus open approach in the RP group are provided in Table 2. No significant differences were observed between the two approaches with respect to the outcome variables except pain scores were higher in the robotic group (2.4 ± 1.7 vs. 0.8 ± 0.8).

**Table 2:** Comparison of clinical characteristics and intra- and postoperative outcomes according to previous operative approach in the RP group *

	Robotic RP (*n* = 21)	Open RP (*n* = 5)	*P* value
Age, years, mean ± SD	67.4 ± 11.2	69 ± 10.9	0.77
ASA score, n (%)			0.34
1	7 (33.3)	3 (60)	
2	14 (66.7)	2 (40)	
3	0 (0)	0 (0)	
BMI, kg/m^2^, mean ± SD	25.3 ± 2.8	26.6 ± 2.6	0.38
Operative time, min, mean ± SD	154.3 ± 57.9	162 ± 34.2	0.71
Conversion, n (%)	5 (23.8)	1 (20)	> 0.99
Hospital stay, days, mean ± SD	1.6 ± 0.9	1.4 ± 0.9	0.64
Intraoperative Complications, n (%)	0 (0)	1 (20)	0.19
Postoperative Complications, n (%)	1 (4.8)	1 (20)	0.35
Return to daily life, days, mean ± SD	3 ± 2.0	2.4 ± 1.1	0.39
Postoperative pain score in 24 h, mean ± SD	2.4 ± 1.7	0.8 ± 0.8	0.01
Readmission, n (%)	0 (0)	1 (20)	0.19
Recurrence, n (%)	0 (0)	0 (0)	
Follow up time, months, mean ± SD	51.3 ± 43.0	35.3 ± 27.5	0.33

*ASA* American Society of Anesthesiologists, *BMI* body mass index

* One laparoscopic case was excluded from the analysis

## Discussion

In this case-match study, we evaluated the feasibility and safety of laparoscopic TEP repair for inguinal hernia in patients post-RP procedure versus those with no history of RP. Compared to patients with no such history, the outcome parameters including intra- and postoperative complications, pain scores, length of hospital stay, time to return to daily life, readmissions and recurrences in post-RP patients were similar. Despite the presence of preperitoneal adhesions which complicate the procedure, this study showed that nearly 17% of 47 inguinal hernia repairs required conversion thus the majority of patients benefited from the advantages of TEP procedure.

Once considered as a contraindication to laparoscopic inguinal hernia repair, history of previous abdominal operations, including appendectomy, cystectomy, prostatectomy and colorectal surgery is no longer considered as a barrier with increasing experience and the advancements in minimally invasive surgery [5, 14–16]. TAPP and TEP techniques are the well-known minimally invasive procedures for inguinal hernia repair. However, considering patients with previous abdominal surgery, the literature shows that TAPP technique is the preferred option by most surgeons [17]. On the other hand, the TEP technique has its own advantages over TAPP such as less risk of visceral injuries, lower incidence of scrotal edema and trocar site hernia and reduced postoperative pain, all of which have been reported to be associated with better patient outcomes [3, 4, 18]. The optimal minimally invasive approach following previous RP, one of the most adhesion-causing surgery, is yet to be determined according to a comprehensive meta-analysis by Aiolfi et al. [19]. In this meta-analysis, the authors report the subanalysis outcomes based on the TAPP and TEP approaches. According to this study, the rate of seroma (13.4 vs. 2.6%), hematoma (7.3 vs. 0.9%) and conversion to open (2.4 vs. 0%) are higher in the TEP approach whereas intraoperative complications (0.9 vs. 0%) and recurrence (1.6 vs. 1.2%) are higher in the TAPP approach. The rates of chronic pain are similar (1.2 vs. 1.2%). Of note, conducting a thorough subgroup analysis for these procedures was not possible due to incomplete reporting of distinct differences among minimally invasive techniques. Based on these findings, this study advises that experience and the proficiency of surgeon are the key factors in choosing the most appropriate technique [19]. Considering these, we adopted the TEP technique and have routinely performed this procedure in over 750 patients regardless of whether there was a history of previous abdominal surgery.

RP is recognized as a significant risk factor for the occurrence of inguinal hernia [6, 20]. A study by Stranne et al. [21] indicates that there is up to a 20% risk of developing inguinal hernia following this procedure. In addition to this, RP results in unique challenges to TEP repair due to the disrupted anatomy and presence of excessive adhesions in the preperitoneal space [7]. Previous studies which evaluated the effects of different types of lower abdominal surgeries on laparoscopic TEP repair conclude that RP, especially open RP, is associated with increased conversion rates [7, 16, 22, 23]. One can state that iliac lymph node dissection during the RP procedure causes severe adhesion formation in the preperitoneal hypogastric and iliac regions which, in turn, challenge the TEP procedure. In addition to these, post-prostatectomy complications and adjuvant radiation treatment, if any, can further increase the extent of adhesions and fibrosis [24]. In order to avoid complications, we altered the TEP procedure in three aspects; (1) For the initial access, a balloon spacemaker was inserted into the preperitoneal space, (2) We especially placed a laparoscope through the spacemaker and preferred to stay at low inflation, reducing inadvertent bleeding secondary to preperitoneal scarring, and (3) We start the dissection laterally close to the anterior abdominal wall and continue towards the lateral abdominal wall and finally the inguinal space for safe exposition of the disrupted anatomy. Meticulous sharp dissection, and the timely and effective haemostasis are important elements to safely perform difficult TEP procedures.

Two of the important issues that must be addressed in the context of TEP procedure in patients with a history of previous RP are longer operative time and increased conversion rates. Key factors contributing to conversion include extensive peritoneal tears, scar tissue from previous operations and fibrotic reactions that can obscure critical surgical landmarks [23]. This naturally ends up with a difficult and longer operation. In the literature, while some studies report a significant increase in the duration of operation, other studies show similar operation times [5, 9, 23]. Considering conversion, earlier studies found higher conversion rates (24%) in patients with a history of bladder or prostate surgeries [9, 22, 23]. A comprehensive study by Trawa et al. revealed a ten-fold increased risk of bladder or vascular injury which contributed to higher conversion rates [25]. On the contrary, prospective studies have shown that increased experience with the TEP technique is one of the important factors in reducing duration of operation and conversion rates [26, 27]. A very recent meta-analysis of minimally invasive surgery following RP has shown that operative time is increased by 21.25 min and conversion rates are higher in the TEP technique [10]. In the present study, the RP group had longer operative time (160 vs. 94 min) and an increased conversion rate (11.1% to TAPP and 14.8% to open surgery vs. none). Expectably, the longer operative times in this group were due to the two factors; extra time spent for the dissection of preperitoneal adhesions and a higher rate of conversions. The sole reason for all the conversions was altered anatomy with the presence of excessive adhesions and fibrosis which did not allow safe dissection in the preperitoneal area. All the cases that did require conversion were free from complications. Notably, there was a substantial decrease in the conversion rate from 50% in the years from 2013 to 2016 to 14% from 2020 to 2024, and no conversion occurred in the last 12 consecutive cases in the RP group after 2022. This finding shows that experience and technique are the keys to success and supports the results reported by Le Page et al. [26] and Dulucq et al. [27]. With respect to all the other outcomes, there were no significant differences between the study groups. Twenty-seven patients in RP group, the TEP approach was attempted, with a successful completion rate of 74.1% without converting to TAPP or open techniques.

Postoperative pain is another critical factor to consider when evaluating inguinal hernia repair techniques. According to Krishna et al. [18], pain levels are three times higher in patients undergoing TAPP repair compared to those undergoing TEP repair in the short term. A prospective study by Andersson et al. has shown that patients who had open surgery experienced greater pain compared to those who opted for the TEP technique [28]. There is also a potential influence of tacks for mesh fixation on postoperative pain and non-fixation methods can be considered in laparoscopic hernia repair. The European Hernia Society (EHS) 2018 guidelines states that no fixation is recommended in all hernia types in TAPP and TEP repair except large direct hernias. Considering the risk of postoperative pain due to traumatic fixation devices, the use of glue fixation should be considered in open and laparo-endoscopic repair [29]. In our practice, we still routinely apply mesh fixation with tacks as a preventive measure to minimize the risk of mesh migration and hernia recurrence, and the main reason for choosing tacks is cost effectiveness. In our analysis, only 13% of the patients reported moderate pain while the vast majority experienced either very low levels of pain or no pain. Moreover, in the RP group, the NRS pain score was lower without being significantly different. Only one patient in non-RP group expressed severe pain. This reduction in pain in RP group could be linked to the disruption of nervous tissue in the dissection area during prostatectomy as also noted in a previous study by Trawa et al. [25].

In the literature, there is not enough information regarding the effect of surgical approach for previous RP on the TEP procedure [26]. In a study, Zuiki et al. [16] claim that open RP is associated with more conversions due to midline incision. In a different study comparing the effects of different surgical approaches for RP on TEP hernia repair, Watt et al. found no major difference among the surgical approaches [9]. In our study, the surgical approach for the majority of RP was robotic (21 patients), followed by open (5 patients) and laparoscopy (1 patient). Excluding the 1 laparoscopic case, the subgroup analysis indicates that TEP procedure can be performed with similar outcomes regardless of whether the previous approach for RP was open or robotic. Therefore, the comparable successful outcomes observed in both robotic and open RP groups may not be attributed to the reduced formation of adhesions associated with minimally invasive surgical techniques as claimed by Stranne et al. [30]. This can also be linked to the high experience of the surgical team. Nevertheless, we found that patients in robotic RP subgroup expressed more pain. It is noteworthy that these findings should be interpreted cautiously due to the small number of patients.

Of note, the presented study is the first study to utilize a propensity score case-matching analysis specifically for TEP repair in post-prostatectomy cases. Along with the other case-match criteria we especially included the year of TEP surgery since this parameter reflects the experience of surgical team with the TEP approach, and therefore, is an important factor to correctly interpret the findings in this type of comparative studies.

Limitations of the present study include its retrospective design and small patient population. However, it gains value from propensity-score matching since the year of surgery was included as one of the matching criteria, which reflects the surgical experience. Additionally, focusing on a single center helps eliminate surgical variability, making the results more reliable and applicable.

## Conclusion

Despite the potential for an increased conversion rate and operative time, laparoscopic TEP inguinal hernia repair is a safe and effective option for patients post-radical prostatectomy. Therefore, previous RP should not be considered a contraindication to TEP technique. Further experience with refined techniques and strategies may help reduce conversion rates in this patient population.
